# Leptin Is an Anti-Apoptotic Effector in Placental Cells Involving p53 Downregulation

**DOI:** 10.1371/journal.pone.0099187

**Published:** 2014-06-12

**Authors:** Ayelén Rayen Toro, Julieta Lorena Maymó, Federico Matías Ibarbalz, Antonio Pérez Pérez, Bernardo Maskin, Alicia Graciela Faletti, Víctor Sánchez Margalet, Cecilia Laura Varone

**Affiliations:** 1 Departamento de Química Biológica, Instituto de Química Biológica de la Facultad de Ciencias Exactas y Naturales, Facultad de Ciencias Exactas y Naturales, Universidad de Buenos Aires, Buenos Aires, Argentina; 2 Departamento de Bioquímica Médica y Biología Molecular, Hospital Universitario Virgen Macarena, Facultad de Medicina, Universidad de Sevilla, Sevilla, España; 3 Hospital Nacional Profesor Alejandro Posadas, Buenos Aires, Argentina; 4 Centro de Estudios Farmacológicos y Botánicos, Facultad de Medicina, Universidad de Buenos Aires, Buenos Aires, Argentina; Innsbruck Medical University, Austria

## Abstract

Leptin, a peripheral signal synthetized by the adipocyte to regulate energy metabolism, can also be produced by placenta, where it may work as an autocrine hormone. We have previously demonstrated that leptin promotes proliferation and survival of trophoblastic cells. In the present work, we aimed to study the molecular mechanisms that mediate the survival effect of leptin in placenta. We used the human placenta choriocarcinoma BeWo and first trimester Swan-71 cell lines, as well as human placental explants. We tested the late phase of apoptosis, triggered by serum deprivation, by studying the activation of Caspase-3 and DNA fragmentation. Recombinant human leptin added to BeWo cell line and human placental explants, showed a decrease on Caspase-3 activation. These effects were dose dependent. Maximal effect was achieved at 250 ng leptin/ml. Moreover, inhibition of endogenous leptin expression with 2 µM of an antisense oligonucleotide, reversed Caspase-3 diminution. We also found that the cleavage of Poly [ADP-ribose] polymerase-1 (PARP-1) was diminished in the presence of leptin. We analyzed the presence of low DNA fragments, products from apoptotic DNA cleavage. Placental explants cultivated in the absence of serum in the culture media increased the apoptotic cleavage of DNA and this effect was prevented by the addition of 100 ng leptin/ml. Taken together these results reinforce the survival effect exerted by leptin on placental cells. To improve the understanding of leptin mechanism in regulating the process of apoptosis we determined the expression of different intermediaries in the apoptosis cascade. We found that under serum deprivation conditions, leptin increased the anti-apoptotic BCL-2 protein expression, while downregulated the pro-apoptotic BAX and BID proteins expression in Swan-71 cells and placental explants. In both models leptin augmented BCL-2/BAX ratio. Moreover we have demonstrated that p53, one of the key cell cycle-signaling proteins, is downregulated in the presence of leptin under serum deprivation. On the other hand, we determined that leptin reduced the phosphorylation of Ser-46 p53 that plays a pivotal role for apoptotic signaling by p53. Our data suggest that the observed anti-apoptotic effect of leptin in placenta is in part mediated by the p53 pathway. In conclusion, we provide evidence that demonstrates that leptin is a trophic factor for trophoblastic cells.

## Introduction

Apoptosis is a naturally occurring event in placental cells. It plays an important role in placenta growth, turnover, senescence and parturition. Apoptotic mechanisms are also associated with the fusion of cytotrophoblast and the differentiation to multinucleate syncytium. Regulators of apoptosis are now considered to have a major role in maintaining the integrity of villous trophoblast [1]. In pregnancies complicated by pre-eclampsia [2], [3] and intra-uterine growth restriction (IUGR) [4], apoptosis is increased in villous trophoblast and are associated with increased formation of syncytial knots [5]. In placental villi, cell turnover is tightly regulated and apoptosis may be induced following cell damage as result of hypoxia or oxidative stress.

The p53 protein is a master transcription factor that increases in response to different stress stimuli such as heat shock, hypoxia, osmotic shock and DNA damage, leading to growth arrest, apoptosis and DNA repair [6]. Upon these cellular stresses, p53 is phosphorylated and acetylated at multiple sites to activate downstream target genes [7], protein levels of p53 are negatively regulated by MDM2, an E3 ubiquitin ligase, via a negative feedback loop that is essential in determining cell survival [8]. Phosphorylation of p53 at Ser-15 leads to the dissociation of MDM2, and p53 degradation is inhibited [9]. It was previously shown that phosphorylation of Ser-46 on p53 contributes to the expression of p53-regulated apoptosis-inducing protein 1 (p53AIP1) [10]. Ser-46 phosphorylation also contributes to the preferential transactivation of other pro-apoptotic genes [11]. Amongst its many functions, p53 promotes transcription of p21, a cell cycle inhibitor, and BAX, a pro-apoptotic mitochondrial pore protein [12].

Another family of proteins, the BCL-2, function as major regulator of the intrinsic apoptotic pathway [13]. Following a death stimulus, the multi-domain pro-apoptotic family members, BAX and BAK proteins form homo- and hetero-oligomers triggering mitochondrial outermembrane permeability and the release of inter-membrane space proteins such as Cytochrome *c* and activation of the downstream apoptotic pathway [14]. The effects of BAX are attenuated by the anti-apoptotic BCL-2 [15]. The BH3-only family members serve as sensors of cellular damage. As result of posttranslational modifications these proteins translocate to the mitochondria, where they activate BAX and BAK. BID is a BH3-only pro-death BCL-2 family molecule that has the ability to interact with the multi-domain pro-death molecules BAX or BAK. This feature constitutes the basis of how the BH3-only molecules may induce apoptosis by either inactivating the anti-death molecules and/or directly activating a multi-domain pro-death molecule [16].

All these factors present in the villous trophoblast indicate a potential role in regulating cell turnover [17]. The study of the molecular mechanisms that regulate placental cell death is important for understanding normal development and a variety of diseases of the placenta.

Leptin, a small non-glycosilated pleiotropic peptide of 146 aminoacid residues (16 kDa), was found to be secreted by adipose tissue [18]. Leptin modulates satiety and energy homeostasis [19]. Evidence shows leptin has a key role implantation and embryo development [20], [21], [22]. The synthesis and secretion of leptin and its functional receptors by trophoblast cells have been widely demonstrated [23], [24], suggesting leptin may act through a paracrine or autocrine mechanism. Plasma leptin concentrations are significantly elevated in pregnant women as compared with those in age and body mass index-matched nonpregnant women [23], [25], and drops sharply after delivery [26]. The expression of leptin in the placenta is tightly regulated by hCG, cAMP and 17β-estradiol [27], [28], [29], [30]. Deregulation of leptin metabolism and/or leptin function in the placenta may be implicated in the pathogenesis of various disorders during pregnancy, such as recurrent miscarriage, gestational diabetes, intrauterine growth restriction, and preeclampsia [31], [32].

Previous results of our group have demonstrated that leptin stimulates the process of proliferation and protein synthesis, and inhibits apoptosis in human trophoblastic cells [33], [34], [35], [36], [37]. In this work we aimed to study the mechanisms of leptin effect on trophoblast survival. We analyzed the expression of some regulators of cell apoptosis and found that leptin decreased the cleavage of Caspase-3 and Poly [ADP-ribose] polymerase 1 (cPARP-1) proteins. On the other hand leptin increased the BCL-2/BAX relationship and diminished the level of Bid. We analyzed p53 protein expression, the master key regulator of death signaling. We found that leptin produced a reduction in p53 level. Moreover we determined a decrease in the phosphorylation of Ser-46 p53 that plays a pivotal role for apoptotic signaling by p53. All these results reinforce the notion of an anti-apoptotic effect of leptin on trophoblastic cells and unravel some of the mechanisms involved.

## Materials and Methods

### Ethics Statement

Written informed consent was obtained from all subjects and all study procedures were approved by ethical review committees at the Virgen Macarena University Hospital and the Alejandro Posadas National Hospital (Bioethics Committee “Dr. Vicente Federico del Giudice”)

### Cell culture and treatments

The human choriocarcinoma cell line BeWo was purchased from the American Type Culture Collection (ATCC, Rockville, MD).The human cytotrophoblastic cell line Swan-71 was generously provided by Dr. Gil Mor (Yale University School of Medicine, New Haven, USA). They were generated by the introduction of human telomerase reverse transcriptase, for the immortalization of primary human cells [38], [39]. BeWo and Swan-71 trophoblastic cells were grown in 45% Dulbecco's modified Eagle medium (DMEM) and 45% HAM F-12 (Invitrogen) supplemented with 10% fetal bovine serum (FBS), 100 U/ml penicillin, 100 µg/ml streptomycin, 2 mM glutamine (Invitrogen), and 1 mM sodium pyruvate (Sigma Chemical Company, St. Louis, MO) at 37°C in 5% CO2.

After 24 h of plating, cell culture medium was replaced with DMEM-F12 - 0% FBS to induce apoptosis and cells were treated for 72 h for total protein immunoblotting or during 24h for protein phosphorylation determinations, in the absence or presence of different concentrations of leptin (from 5 ng/ml to 250 ng/ml). Cells cultured continuously with 10% FBS were used as control. In experiments designed to diminish endogenous leptin expression, an antisense oligonucleotide (AS), complementary to the first five amino acids of leptin mRNA sequence [18] was used. The sequence is: 5′GCACAGGGTTCCCCAATGCAT3′. Different concentrations of AS from 1 µM to 4 µM were used during 72 h incubations as reported previously [33]. Then cells were washed with PBS and solubilized in lysis buffer (1× phosphate-buffered saline (PBS), 1% Nonidet P-40, 0.5% sodium deoxycholate, 0.1% sodium dodecyl sulfate (SDS), and 10 mg/ml phenylmethanesulfonyl fluoride (PMSF)). Cell lysates were used for Western blot analysis.

### Placental explants collection and processing

Human placentas (n = 5) were obtained after vaginal delivery following normal term pregnancies and immediately suspended in ice-cold PBS and transported to the laboratory, where they were washed 2–3 times in sterile PBS to remove excess blood. Villous tissue free of visible infarct, calcification, or hematoma was sampled from at least five cotyledons at a distance midway between the chorionic and basal plates. These core parts of cotyledons were cut into multiple cubic segments (10–15 mg wet weight) and thoroughly rinsed with cold Hanks medium pH 7.4 (137 mM NaCl, 5 mM KCl, 1 mM CaCl_2_, 1 mM MgSO_4_, 0.3 mM Na_2_HPO_4_, 0.4 mM KH_2_PO_4_, and 4 mM NaHCO_3_). None of the donor patients suffered from anomalous pregnancy.

### Treatments of placental explants

Placental explants for all experiments were treated only during 24 h as they represent a more physiologic model and shorter times are needed to evidence leptin effect.

Placental explants were randomly distributed in tubes containing 1 ml of Hanks medium (n = 1 explant/tube, 4 replicates per treatment), maintained in a shaking water bath at 37°C during 5 min to equilibrate temperature and incubated for 24 h in DMEM-F12 0% FBS in the absence or presence of different concentrations of leptin (from 5 ng/ml to 250 ng/ml). Explants were removed from the bath, centrifuged for 2 min at 2000 g at 4°C and resuspended in 500 µl of lysis buffer (1× PBS, 1% Nonidet P-40, 0.5% sodium deoxycholate, 0.1% SDS, and 10 mg/ml PMSF) during 30 min at 4°C on an orbital shaker and later centrifuged at 10000 g for 20 min. Supernatants were analyzed by Western blot.

### Western blot analysis

Total cell lysates were prepared in lysis buffer. The lysates were centrifuged at 10000 g for 10 min to remove cellular debris. The protein concentration of the supernatant was determined by the Bradford staining method [40], with bovine serum albumin (BSA) as standard. Lysates were mixed with Laemmli's sample buffer containing 2% SDS and 30 mM β-mercaptoethanol, boiled for 5 min, resolved by SDS-PAGE on a 12% gel, and electrophoretically transferred to a nitrocellulose membrane (Hybond, Amersham Pharmacia). Membranes were equilibrated in 1× PBS and non-specific binding sites were blocked by 5% non-fat milk in PBS at room temperature for 1 h. The membranes were then immunoblotted with monoclonal mouse anti-Caspase-3 (1∶1000, Cell Signaling), monoclonal rabbit anti- leptin (1∶1000, Santa Cruz), monoclonal rabbit anti-PARP (1∶100, Santa Cruz), monoclonal mouse anti-p53 (1∶1000, Santa Cruz), polyclonal rabbit anti-phospho Ser-46 p53 (1∶1000, Cell Signaling), polyclonal rabbit anti-BID (1∶1000, Cell Signaling), polyclonal rabbit anti-BAX (1∶1000, Santa Cruz), or polyclonal rabbit anti-BCL-2 (1∶1000, Epitomics). Loading controls were performed by immunoblotting the same membranes with polyclonal rabbit anti-β-actin (1∶1000, Santa Cruz), monoclonal mouse anti-α-tubulin (1∶2500, Santa Cruz) or monoclonal mouse anti glyceraldehyde-3-phosphate dehydrogenase (GAPDH) (1∶2500, Calbiochem). The antibodies were detected using horseradish peroxidase-linked goat anti-rabbit IgG (1∶10000, Santa Cruz) or goat anti-mouse IgG (1∶10000, Santa Cruz) and visualized by the Amersham Pharmacia ECL Chemiluminescence signaling system and a Bio-Imaging Analyzer Fujifilm LAS-1000. Quantification of protein bands was determined by densitometry using Image J 1.47 software (Wayne Rasband National Institute of Health, USA).

### DNA fragmentation assay

Apoptosis was evaluated by examining the characteristic pattern of DNA laddering to assay DNA fragmentation. Placental explants (40 mg of tissue per treatment) were incubated in 1 ml of DMEM-F12 in the presence or absence of FBS containing 100 ng/ml of leptin in a shaking water bath at 37°C. After 24 h of treatment, placental explants were harvested, washed in PBS and homogenized in 500 µl of lysis buffer containing 1% of SDS, 50 mM EDTA, 50 mM Tris HCl and 50 mM NaCl. Then, proteinase K (10 mg/mL) was added to the homogenates and incubated at 55°C during 2 hours. Afterwards, 5 M NaCl was added, mixed by inversion and centrifuged at 15000 rpm for 15 min at 4°C. Supernatants were transferred and cold ethanol was added, mixed by inversion and the mixtures were left at −20°C overnight. Samples were centrifuged at 15000 rpm for 15 min at 4°C and DNA pellet was air dried and resuspended in 100 µL of sterile water. DNA fragments were separated by a 2% agarose gel electrophoresis at 80 V for 1.5 h. The gels were stained with 0.5 mg/ml ethidium bromide and low DNA fragments visualized on a UV-illuminator. Quantification of low molecular weight bands was determined by densitometry using Image J 1.47 software.

### Plasmids

We used a vector derived from pGL2 with Bax promoter region cloned upstream luciferase reporter gene (pBax-Luc). It was kindly provided by Dr. Susana Llanos (CNIO, Madrid, Spain). To normalize the efficiency of individual transfections, pRSV-βgal containing the β-galactosidase gene under the control of the Rous sarcoma virus (RSV) was used. To perform transient transfection assays, plasmids were purified using the Midipreps Wizard kit (Promega Co.), and DNA concentration was estimated spectrophotometrically.

### Transient transfection experiments

For transient transfection experiments, BeWo cells were plated at a density of 2.5×105 cells/ml onto six-well dishes containing 2 ml of DMEM-F12 plus 10% FBS. Cells were incubated for 24 h. Medium was replaced and transfection of cells was performed according to the standard liposome-mediated method. In order to determine the sensitivity of the method in this cell type, a standard dose of reporter plasmid vs. light emission was performed (data not shown). Typically 5 µg of the luciferase reporter and 5 µg of pRSV-βgal internal control construct were transfected using 5 µl of LipofectAMINE (Life Technologies, Inc.). The medium was replaced after 5 h with DMEM-F12 0% FBS with the addition of the different effectors. Transfection analysis was performed by duplicate in each of at least three independent experiments.

### Assays for luciferase and β-galactosidase activities

Luciferase activity in cell lysates was measured using the Luciferase Assay System (Promega). Cells were washed with PBS and harvested 72 h after transfection using 50 µl of lysis buffer. Cell extracts were centrifuged and 30 µl of the supernatant was mixed with 50 µl of luciferase assay buffer. Luciferase activity was measured with the GloMax-Multi+ Microplate Multimode Reader luminometer (Promega Corp). β-galactosidase activity was assayed using 1 mg of *o*-nitrophenyl β-D-galactopyranoside (AmResco) as the substrate in buffer Z (60 mM Na_2_HPO_4_, 40 mM NaH_2_PO_4_, 10 mM KCl, 1 mM MgSO_4_, 0.07% β-mercaptoethanol) and incubated at 37°C until yellow staining. The product was determined by absorption at 420 nm. This value was used to correct variations in transfection efficiency. Luciferase results were calculated as the ratio of luciferase activity per unit of β-galactosidase activity. Duplicate samples were analyzed for each data point.

### Quantitative real-time RT-PCR assay

Abundance of p53 mRNA was determined by quantitative real time RT-PCR reaction (*q*RT-PCR). Total RNA was extracted from placental explants using TRISURE reagent, according to the manufacture's instructions (Bioline Co). Concentration and purity of the isolated RNA were estimated by spectrophotometry at 260 and 280 nm. For cDNA synthesis, 5 µg of total RNA was reverse-transcribed at 50°C during 1 h using the Transcriptor first Strand cDNA synthesis Kit (Roche). Quantitative real time PCR reaction was performed using the following primers based on the sequences of the NCBI GenBank database: p53, forward, 5′GGAAGAGAATCTCCGCAA3′; reverse, 5′AGCTCTCGGAACATCTCGAAG3′; and cyclophilin, forward, 5′CTTCCCCGATACTTCA3′; reverse, 5′TCTTGGTGCTACCTC 3′. *q*RT-PCR Master Mix Reagent kit was obtained from Roche (Fast Start universal SYBR Green) and PCR reactions were performed on a Chromo 4 DNA Engine (BioRad). A typical reaction contained 10 µM of forward and reverse primer, 3 µl of cDNA and the final reaction volume was 25 µl. The reaction was initiated by preheating at 50°C for 2 min, followed by heating at 95°C for 10 min. Subsequently, 41 amplification cycles were carried out as follows: denaturation 15 sec at 95°C and 1 min annealing and extension 1 min at 59°C. The threshold cycle (C_T_), from each well was determined by the Opticon Monitor 3 Program. Relative quantification was calculated using the 2^−ΔΔCT^ method [41]. For the treated samples, evaluation of 2^−ΔΔCT^ indicates the fold change in gene expression, normalized to a housekeeping gene (cyclophilin), and relative to the untreated control.

### Data analysis

Experiments were repeated separately at least three times to assure reproducible results. Results are expressed as the mean ± standard deviation (S.D.). The statistical significance was assessed by ANOVA followed by different post hoc tests indicated in each figure and was calculated using the Graph Pad Instat computer program (San Diego, CA). A *P*-value <0.05 was considered statistically significant.

## Results

### Leptin diminishes apoptosis in placental cells

**Figure 1:**
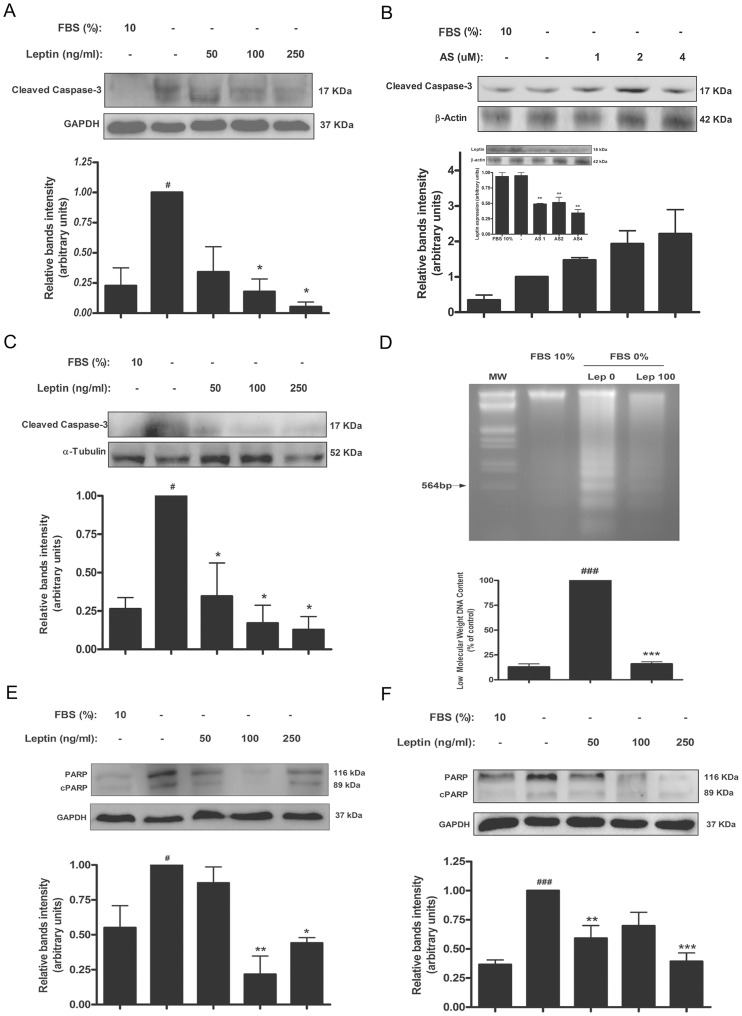
Leptin prevents apoptosis in placental cells. Caspase activity was determined in BeWo cells (A and B) or placental explants (C). A–B) BeWo (1×10^6^ cells) were plated in complete DMEM-F12 media (10% FBS). After 24 h cells were incubated during 72 h in DMEM-F12 0% FBS, with increasing doses of leptin (A) or an oligonucleotide complementary to leptin sequence (AS) (B), as indicated in the figure. Cell extracts were prepared as indicated in Materials and Methods and proteins were separated on SDS-PAGE gels. The inset shows representative blots and quantification confirming reduced leptin expression in AS treated cells. C) Placental explants were processed as described in Material and Methods and incubated in DMEM-F12 0% FBS media supplemented with increasing leptin doses during 24 h. Placental extracts were prepared and proteins were separated on SDS-PAGE gels. Caspase-3 cleaved fragment was determined by Western blot analysis (A, B and C). D) Gel electrophoresis of apoptotic DNA fragmentation. Inspection of electrophoretic profiles revealed a lower ladder formation in presence of leptin. Quantification of low molecular weight bands is shown. DNA fragmentation assay was performed as indicated in Materials and Methods. E) Swan-71 cells (1×10^6^ cells) were plated in complete DMEM-F12 media (10% FBS). After 24 h cells were incubated during 72 h in DMEM-F12 0% FBS, with increasing doses of leptin as indicated in the figure. Cell extracts were prepared as indicated in Materials and Methods and proteins were separated on SDS-PAGE gels. **F)** Placental explants were processed as described in Material and Methods and incubated in DMEM-F12 0% FBS media supplemented with increasing leptin doses during 24 h. Placental extracts were prepared and proteins were separated on SDS-PAGE gels. In both cases (E and F) cleaved PARP (cPARP) fragment was determined by Western blot analysis. Cells cultured with DMEM-F12 media supplemented with 10% FBS were used as control. Molecular weights were estimated using standard protein markers. Molecular mass (kDa) is indicated at the right of the blot. Loading controls were performed by immunoblotting the same membranes with anti-β-actin, anti-α-tubulin or anti-GAPDH. Bands densitometry is shown in lower panels. Results are expressed as mean ± SD for three independent experiments. Statistical analyses were performed by ANOVA and Bonferroni's multiple comparison *post hoc* test, relative to FBS 10% (#) or FBS 0% (*). # p<0.05, ###p<0.001, * p<0.05, ** p<0.01, ***p<0.001.

We previously reported that leptin has a trophic effect in trophoblastic JEG-3 and BeWo cells, preventing the apoptosis promoted by serum deprivation [34]. Apoptosis was investigated by determination of Caspase-3 activated form and cPARP by Western blot and DNA fragmentation assay. Previously we found that leptin prevents Caspase-3 activation in JEG-3 trophoblastic cells involving MAPK pathway [34]. As it is shown in Figure 1A, serum starvation signal is sufficient to increase Caspase-3 activation in BeWo cells. Treatment with leptin reduced Caspase-3 activation in a dose dependent manner. Maximal effect was achieved at 250 ng leptin/ml. Since BeWo cell line expresses leptin [33], we speculated that perhaps the Caspase-3 activation effect exerted by exogenous leptin could be partially masked by endogenous production. To address this issue, BeWo cells were treated with different concentrations of leptin AS. As seen in Figure 1B, treatment with 1–4 µM AS increased Caspase-3 activation. In Figure 1C results obtained in human placental explants, a more physiologic model, are shown. As seen, leptin treatment lowered Caspase-3 activation. Leptin effect on late apoptosis was determined in placental explants using a different experimental approach, the DNA fragmentation assay. With this technique it is possible to determine the presence of low DNA fragments, products from apoptotic DNA cleavage. As it is seen in Figure 1D, serum deprivation increased the apoptotic cleavage of DNA meanwhile 100 ng leptin/ml significantly reduced low weight population of DNA fragments.

PARP cleavage is catalyzed by activated Caspase-3 which prevents PARP-mediated DNA repair processes. To further study leptin effect on Caspase-3 activity, the p89 fragment of cleaved PARP-1 (cPARP) was determined. Serum deprivation significantly raised cPARP and treatment with leptin decreased it in trophoblastic cells (Fig. 1E) and placental explants (Fig. 1F). Taken together these results highlight the survival effect exerted by leptin on placental cells.

### Leptin enhances BCL-2/BAX relationship in placental cells probably by augmenting BCL-2 expression

**Figure 2:**
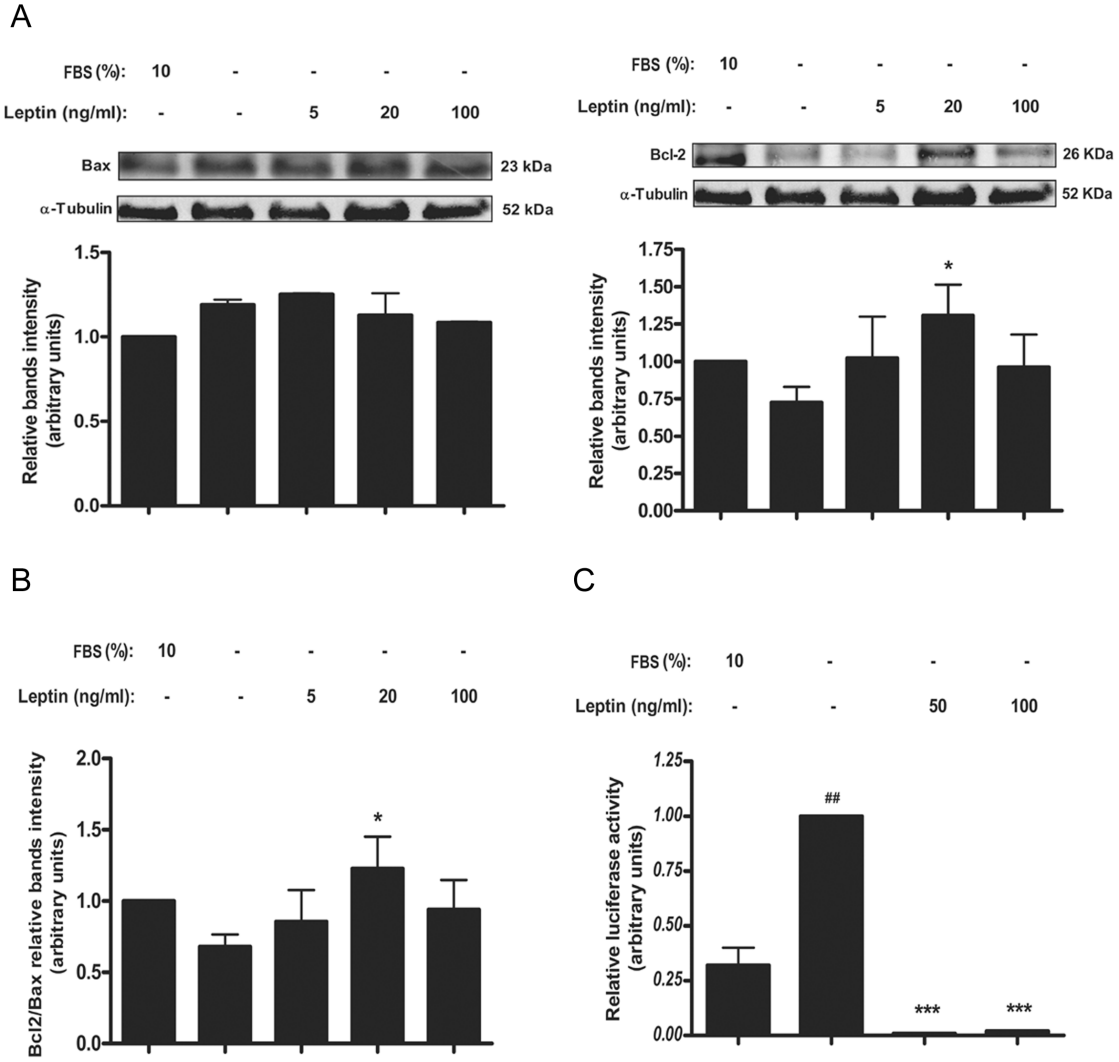
Leptin enhances BCL-2/BAX relationship in placental cells. A) Swan-71 cells (1×10^6^ cells) were plated in DMEM-F12 media in the absence of serum and incubated during 72 h with different doses of leptin. DMEM-F12 10% FBS was used as a control. Cell extracts were prepared as indicated in Materials and Methods. Proteins were separated on SDS-PAGE gels and BCL-2 and BAX expression was determined by Western blot analysis. Molecular weights were estimated using standard protein markers. Molecular mass (kDa) is indicated at the right of the blot. Loading controls were performed by immunoblotting the same membranes with anti-α-tubulin. Bands densitometry is shown in lower panels, results are expressed as mean ± SD for three independent experiments. B) Leptin increased BCL-2/BAX relationship. C) BeWo cells were transiently transfected with a plasmid containing a section of BAX promoter (pBax-Luc). After transfection, cells were incubated for 72 h in DMEM-F12 and treated with increasing leptin doses. Cell extracts were prepared as indicated in Materials and Methods and Luciferase activity was normalized to β-galactosidase activity. Activity obtained in the absence of leptin and pBax-Luc was set as control. Statistical analyzes were performed by ANOVA. Asterisks indicate significant differences from the control according to Bonferroni's multiple comparison *post hoc* test, relative to FBS 10% (#) or FBS 0% (*). ## p<0.01, * p<0.05, *** p<0.001.

**Figure 3:**
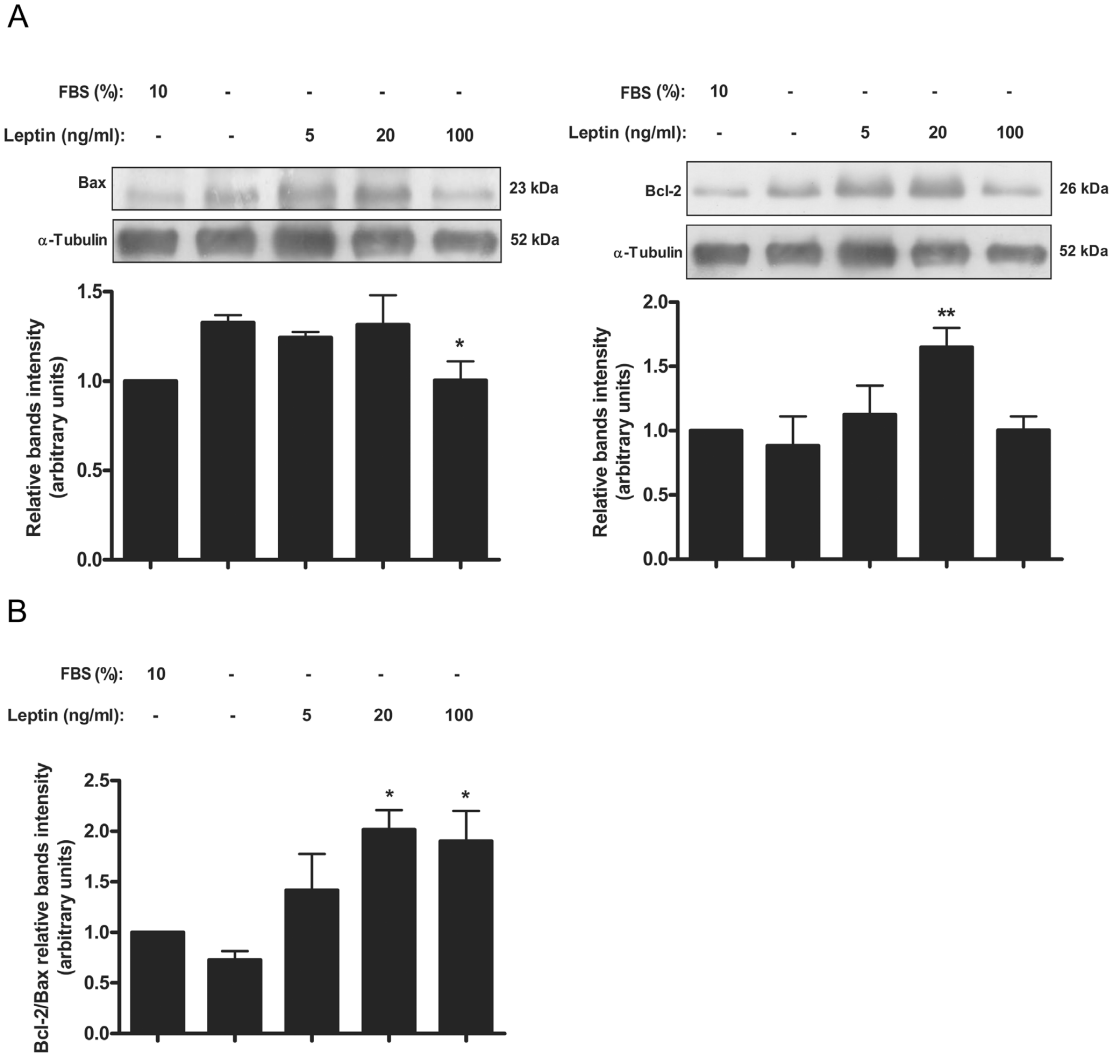
Leptin enhances BCL-2/BAX relationship in human placental explants. A) Placental explants were processed as described in Materials and Methods and incubated during 24 h in DMEM-F12 media supplemented with increasing leptin doses. DMEM-F12 10% FBS was used as control. Placental extracts were prepared and proteins were separated on SDS-PAGE gels. BCL-2 and BAX expression were determined by Western blot analysis as indicated in the Figure. Molecular weights were estimated using standard protein markers. Molecular mass (kDa) is indicated at the right of the blot. Loading controls were performed by immunoblotting the same membranes with anti-α-tubulin. Bands densitometry is shown in lower panels, results are expressed as mean ± SD for three independent experiments. B) BCL-2/BAX relationship is shown. Statistical analyzes were performed by ANOVA. Asterisks indicate significant differences from the control according to Bonferroni's multiple comparison *post hoc* test, relative to FBS 0% (*). * p<0.05, ** p<0.01

BCL-2 and BAX expression, an anti- and a pro- apoptotic member of the BCL-2 family respectively, was analyzed in cell culture (Fig. 2) and placental explants (Fig. 3) stimulated with different leptin concentrations to further characterize apoptotic effect of leptin in trophoblastic cells. Serum starvation reduced BCL-2 level in Swan-71 cells measured by Western blot analysis (Fig. 2A). Leptin treatment at 20 ng leptin/ml significantly augmented BCL-2 levels in Swan-71 cells (Fig. 2A) and placental explants (Fig. 3A).

Serum starvation increased BAX levels in Swan-71 cells and placental explants (Figs. 2A and 3A). Leptin decreased BAX levels, at least in primary placental explants, with a maximal effect at 100 ng leptin/ml (Fig. 3A). In Figures 2B and 3B it could be seen that 20 ng leptin/ml increased BCL-2/BAX relationship. We next analyzed the possibility that leptin might modulate BAX expression at the transcriptional level. BeWo cells were transiently transfected with pBax- Luc reporter construct and treated with different leptin concentrations. As displayed in Figure 2C, incubation of cells with serum depleted media enhanced BAX gene expression and treatment with 50-100 ng leptin/ml significantly reduced it.

### Leptin diminishes BID level in placental cells

**Figure 4:**
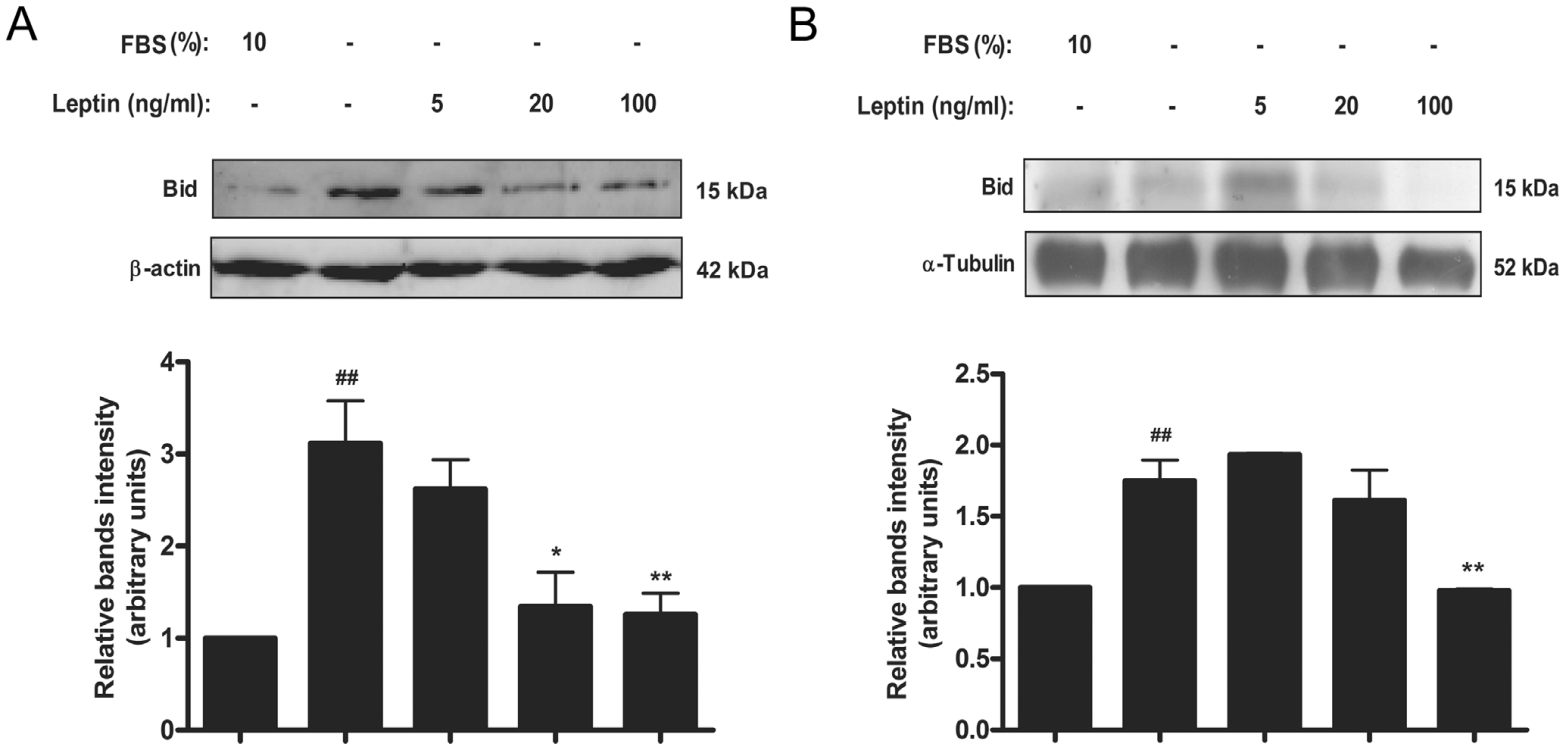
Leptin diminishes BID level in placenta. A) Swan-71 cells (1×10^6^ cells) were plated in DMEM-F12 media 10% FBS. After 24 h, cells were incubated during 72 h with increasing doses of leptin in DMEM-F12 0% FBS. Cell extracts were prepared as indicated in Materials and Methods and proteins were separated on SDS-PAGE gels. B) Placental explants were processed as described in Material and Methods and incubated in DMEM-F12 0% FBS media supplemented with increasing leptin doses during 24 h. Placental extracts were prepared and proteins were separated on SDS-PAGE gels. In both cases (A and B) BID cleaved fragment was determined by Western blot analysis. DMEM-F12 10% FBS was used as control. Molecular weights were estimated using standard protein markers. Molecular mass (kDa) is indicated at the right of the blot. Loading controls were performed by immunoblotting the same membranes with anti-α-tubulin or anti-β-actin. Bands densitometry is shown in lower panels. Results are expressed as mean ± SD for three independent experiments. Statistical analyzes were performed by ANOVA. Asterisks indicate significant differences from the control according to Bonferroni's multiple comparison *post hoc* test, relative to FBS 10% (#) or FBS 0% (*). ## p<0.01, * p<0.05, ** p<0.01

BH3-only proteins, like BID, are essential for initiation of apoptosis signaling, being activated transcriptionally and/or post-transcriptionally [42]. We decided to analyze leptin effect on BID levels in Swan-71 cells and human placental explants. As it is seen in Figure 4A incubation of the cells with serum free media significantly enhanced BID expression. The treatment with leptin significantly decreased Bid expression. Maximum effect was achieved with a leptin dose of 100 ng/ml. Similar results were obtained when BID expression was determined in human placental explants (Fig. 4B).

### Leptin diminishes p53 level in trophoblastic cells and placental explants

**Figure 5:**
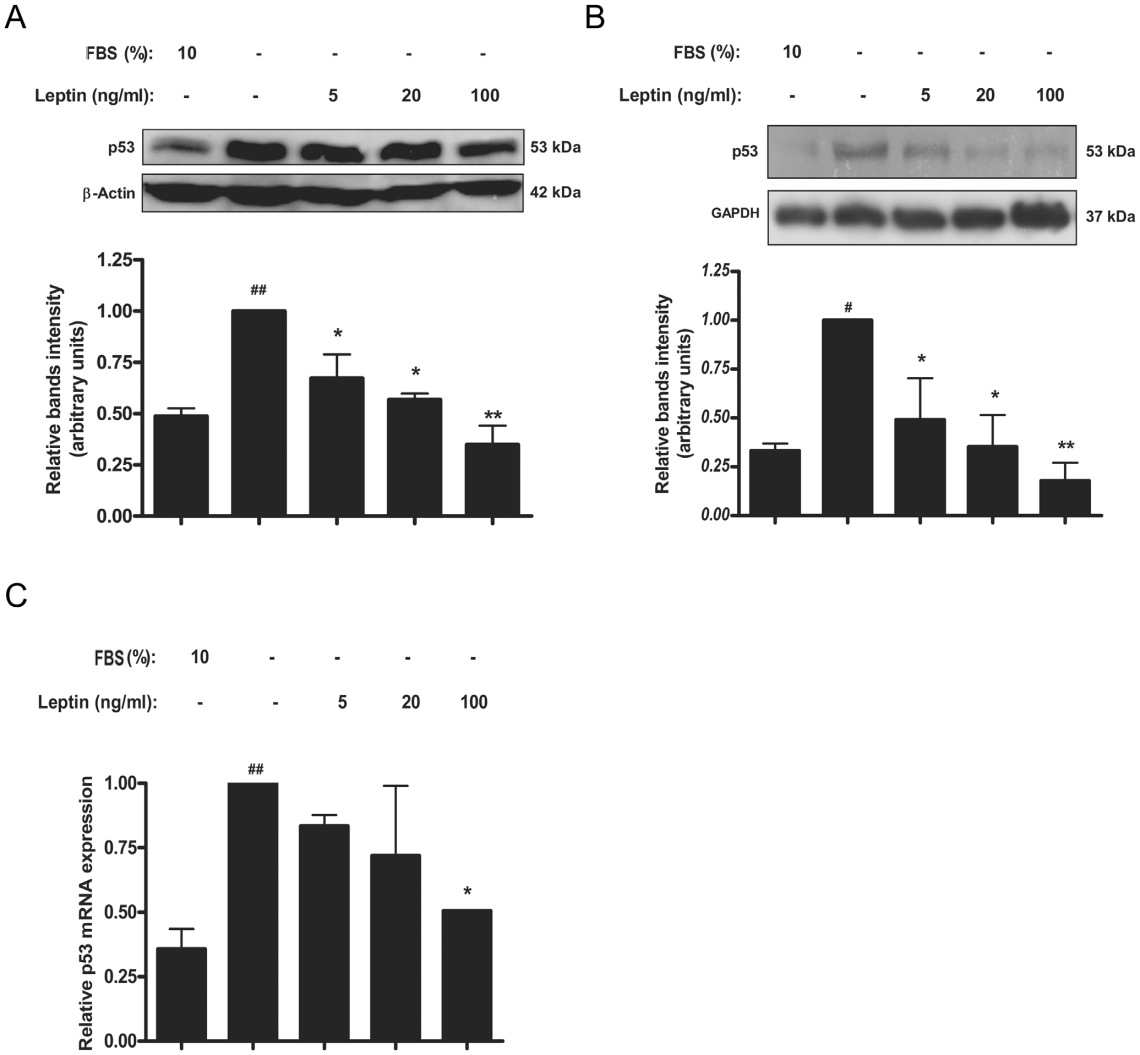
Leptin reduces p53 levels in placenta. A) Swan-71 cells (1×10^6^ cells) were plated in DMEM-F12 media in the absence of serum and incubated during 72 h with different leptin concentrations. Cell extracts were prepared as indicated in Materials and Methods and proteins were separated on SDS-PAGE gels. B) Placental explants were processed as described in Material and Methods and incubated in DMEM-F12 media supplemented with increasing leptin doses during 24 h. Placental extracts were prepared and proteins were separated on SDS-PAGE gels. In both cases (A and B) p53 was determined by Western blot analysis. DMEM-F12 10% FBS was used as a control. Molecular weights were estimated using standard protein markers. Molecular mass (kDa) is indicated at the right of the blot. Loading controls were performed by immunoblotting the same membranes with anti-α-GAPDH. Bands densitometry is shown in lower panels. Results are expressed as mean ± SD for three independent experiments. C) p53 expression in human placental explants was determined by *q*RT-PCR. RNA was extracted as described in Materials and Methods. Statistical analyzes were performed by ANOVA. Asterisks indicate significant differences from the control according to Bonferroni's multiple comparison *post hoc* test, relative to FBS 10% (#) or FBS 0% (*). # p<0.05, ## p<0.01, * p<0.05, ** p<0.01.

The tumor suppressor p53 is best characterized as a transcription factor that binds to specific DNA sequences and transactivates a number of genes with a variety of functions including cell cycle arrest, apoptosis, and others [43]. To determine if leptin modifies p53 levels in trophoblastic Swan-71 cells, p53 expression was analyzed. Serum starvation induced p53 both at mRNA and protein levels (Fig. 5). Leptin treatment significantly reduced p53 protein levels. Maximum effect was achieved at doses of 100 ng/ml (Figure 5A). Human placental explants were also analyzed. As it is shown in Figure 5B, leptin significantly diminished p53 protein levels in a dose dependent manner. Maximum effect was achieved with a dose of 100 ng/ml of leptin. Real time PCR assay was performed to determined leptin effect on p53 mRNA levels, in placental explants. Leptin treatment in serum free media showed a significant dose-dependent reduction of p53 mRNA level (Figure 5C).

### Leptin diminishes p53 Ser-46 phosphorylation in trophoblastic cells and placental explants

**Figure 6:**
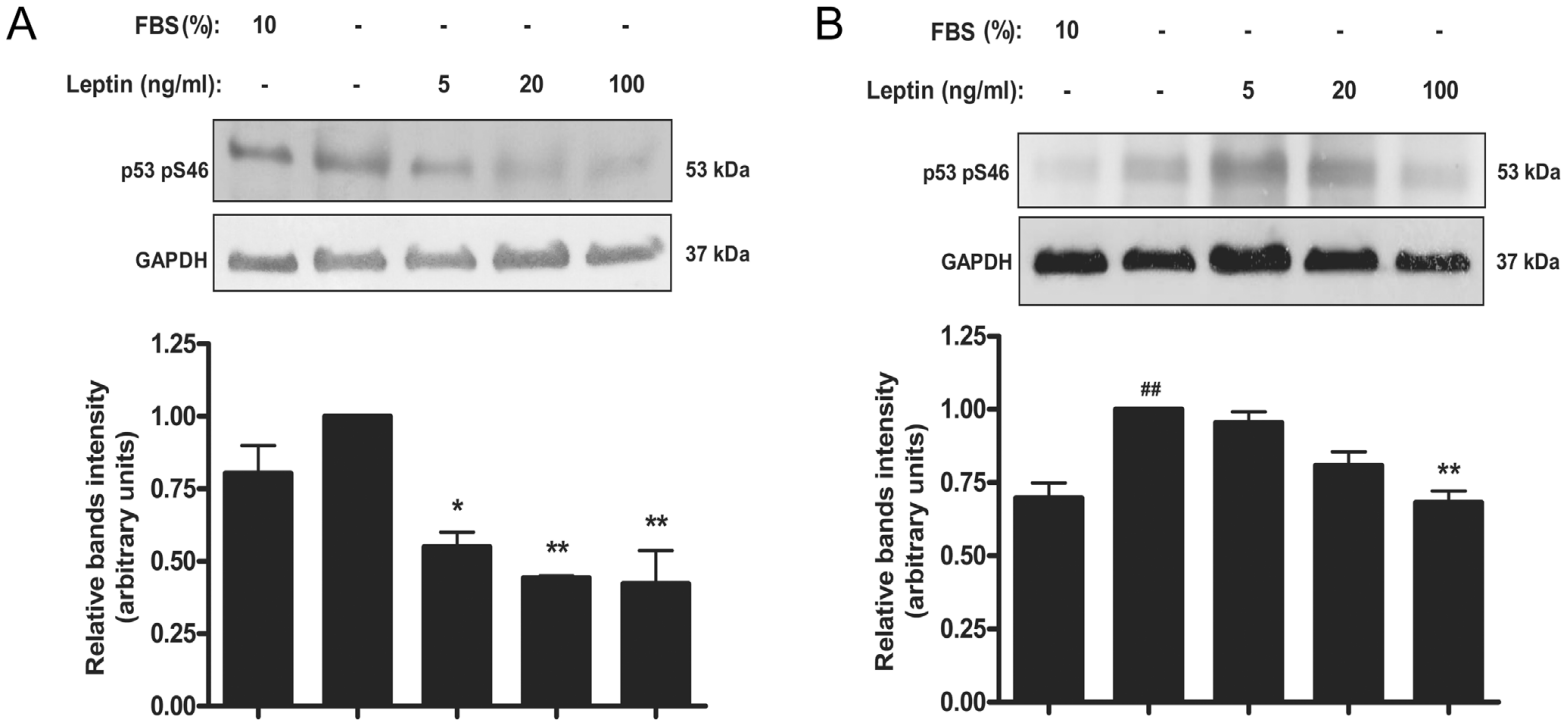
Leptin reduces Ser-46 p53 phosphorylation level in placenta. A) Swan-71 cells (1×10^6^ cells) were plated in DMEM-F12 10% FBS. After 24 h cells were incubated during 24 h with increasing doses of leptin in DMEM-F12 in the absence of serum. Cell extracts were prepared as indicated in Materials and Methods and proteins were separated on SDS-PAGE gels. B) Placental explants were processed as described in Material and Methods and incubated in DMEM-F12 0% FBS media supplemented with increasing leptin doses during 24 h. DMEM-F12 10% FBS was used as a control. Placental extracts were prepared and proteins were separated on SDS-PAGE gels. In both cases (A and B) Phospho Ser-46 p53 was determined by Western blot analysis. DMEM-F12 media supplemented with 10% FBS was used as control. Molecular weights were estimated using standard protein markers. Molecular mass (kDa) is indicated at the right of the blot. Loading controls were performed by immunoblotting the same membranes with anti-GAPDH. Bands densitometry is shown in lower panels. Results are expressed as mean ± SD for three independent experiments. Statistical analyzes were performed by ANOVA. Asterisks indicate significant differences from the control according to Bonferroni's multiple comparison *post hoc* test, relative to FBS 10% (#) or FBS 0% (*). ## p<0.01 * p<0.05, ** p<0.01.

The phosphorylation at Ser-46 was shown to be involved in the regulation of apoptosis. We decided to evaluate the effect of leptin on the phosphorylation of p53 Ser-46. As it is shown in Figure 6A, the absence of serum slightly increased the phosphorylation of Ser-46 in p53. Treatment with leptin significantly decreased the phosphorylation of Ser-46 of p53 with a maximal effect achieved with a leptin dose of 100 ng/ml. Also in human placental explants (Fig. 6B), 100 ng leptin/ml significantly decreased the phosphorylation of p53 Ser-46.

## Discussion

The programmed cell death or apoptosis is an essential process for normal placental development but is highly increased in placental diseases as hydatidiform mole, pre-eclampsia and intra-uterine growth restriction (IUGR) [44]. During normal pregnancy, trophoblast apoptosis increases while placenta grows. As mononucleated villous trophoblast cells proliferate, some daughter cells differentiate and fuse forming the syncytiotrophoblast. The maintenance and integrity of the syncytial layer depend on the continuous input of new material. Old and no longer needed cellular material is shed from the apical surface of the syncytiotrophoblast and released into the maternal circulation as apoptotic syncytial knots [45].

Trophoblast apoptosis, as other types of cell apoptosis, includes the extrinsic and intrinsic pathways culminating in the activation of caspases. The formation of apoptotic bodies and liberation of this material into the maternal circulation was demonstrated and may be detected in maternal circulation during the course of normal gestation [44]. Even though placental apoptosis is a normal process during pregnancy it may be initiated by different damaging stimuli, including hypoxia and oxidative stress [46]. Excessive placental trophoblast apoptosis and shedding of placenta debris are associated with different placental-insufficiency-related pregnancy's complications, and some molecular biomarkers are being studied for their potential in diagnosis of these pathologies [47]. It is postulated that placental-derived material might induce maternal endothelial dysfunction of pre-eclampsia [48]. Widespread apoptosis of the syncytiotrophoblast may also impair trophoblast function leading to the reduction in nutrient transport seen in IUGR.

However, during normal pregnancy, extravillous trophoblast cells invade maternal uterine tissues. The interstitial trophoblast penetrates decidual tissues reaching the inner third of the myometrium. A subset of the interstitial trophoblast, transforms uterine spiral arteries into large-bore conduits to enable the adequate supply of nutrients and oxygen to the placenta and thus to the fetus. Controlled invasion by trophoblast of uterine decidua, myometrium and spiral arteries, is essential for normal fetoplacental development. The balance between trophoblast apoptosis and proliferation represents a mechanism to control normal trophoblast invasion [49]. In this regard leptin, was described as an important cytokine regulating trophoblast survival, promoting growth and preventing the apoptotic process [33]. These effects may be of physiological relevance since trophoblastic cells are an important source of increased leptin production during pregnancy [23], [32]. Moreover, leptin levels are increased under stressful condition for placenta cells such as preeclampsia or gestational diabetes [32], [50]. This overproduction of leptin may be helpful to prevent the stress-mediated apoptosis of the trophoblastic cells. However, little is known about the molecular mechanisms underlying these effects. Leptin activation of MAPK pathway has been previously found to be the mechanism whereby leptin promotes cell survival preventing apoptosis [51], [52]. In trophoblastic JEG-3 cells we have found that leptin prevents the apoptotic process triggered by the deprivation of serum by means of the activation of MAPK pathway [34], but little is known about the mechanisms involved.

In this study, we employed BeWo human choriocarcinoma cells and Swan-71 cells. Human placental explants from healthy donors were also studied to confirm the physiological relevance of the mechanisms involved in leptin survival effect. BeWo cells maintain many characteristics of human trophoblast cells and have been widely used to study placental function [53]. Swan-71 cell line has attributes that are characteristic of primary first trimester trophoblast cells. The Swan-71 cells are positive for the expression of cytokeratin 7, vimentin and HLA-G and exhibit a cytokine and growth factor profile that is similar to primary trophoblast cells [38]. Swan-71 cells also express the long isoform of leptin receptor (unpublished results). They represent a valuable model for *in vitro* trophoblast studies. In this study we confirmed that leptin diminishes apoptosis in placental cells by virtue of the decrease of the Caspase-3 activation both in BeWo cells and in human placental explants. Moreover, when endogenous leptin expression was inhibited using an oligonucleotide complementary to leptin mRNA, cleaved Caspase-3 peptide increased. Leptin also diminished the cleavage of PARP, a nuclear DNA-binding protein that influences DNA repair, DNA replication, modulation of chromatin structure and apoptosis. These results reinforced the notion of leptin as a survival factor. By a different experimental approach, we found that leptin treatment reduced the presence of small fragments product of DNA cleavage. These data prompted us to study the mediators that could be involved in placental cell apoptosis.

It is well known that BCL-2-family proteins are central regulators of cell life and death. The first pro-apoptotic member of the family, BAX (BCL-2 Antagonist X) was identified as a BCL-2-interacting protein that opposed BCL-2 and promoted apoptotic cell death [54]. Subsequent data led to the identification of more pro-apoptotic proteins that contain several conserved regions of sequence similarity including BAX, BAK, and BOK. A variety of genetic and biochemical studies argue that these multidomain proteins are the effectors of the mitochondrial cell death pathway, with anti-apoptotic proteins such as BCL-2 and BCL-xL operating as upstream regulators that oppose the intrinsic death-inducing actions at mitochondrial membranes [55]. We observed that leptin increased the relation BCL-2/BAX both in Swan-71 and human placental explants. The modulation of these ratios by leptin was due principally to an increase in BCL-2 protein. Our data showed also a reduction of BAX protein in placental explants. Similar results were observed in lymphocytes B. Leptin promotes B-cell homeostasis by inhibiting apoptosis through the activation of expression of BCL-2 and inhibits the expression of the pro-apoptotic BCL-2 family proteins, BAX, BIM, and BAD [56]. An upregulation of the BCL-xL gene was also seen in lymphocytes treated with leptin, suggesting that leptin might contribute to the recovery of immune suppression by inhibiting lymphocyte apoptosis [57]. We demonstrated that leptin represses BAX expression at the transcriptional level in BeWo cells as seen by transient transfection reporter assay, suggesting that mechanisms of regulation of gene BAX expression could be involved. We observed that after leptin treatment, BAX protein remained unchanged in Swan-71 cells. The discrepancy observed on leptin effect on BAX expression between BeWo and placental explants and Swan-71 cells might be due to differences in the gestational age of these models. Further studies are in progress at our lab to clarify this fact. The major finding of this study is the increase of BCL-2/BAX ratio caused by leptin. It was reported that an increase of BCL-2 protein might influence the level of BAX protein, but the mechanisms are still under debate. In this report, upon apoptosis induction by IL-3 withdrawal, the over-expression of BCL-2 maintained the high levels of mitochondrial BAX content but Cytochrome *c* release was nevertheless inhibited [58]. This could explain the unchanged BAX expression in Swan-71 cells, when BCL-2 is augmented by leptin treatment.

3D solution structure of BAX in the soluble inactive state indicates that the C-terminal membrane anchoring domain is tucked into the same pocket that binds BH3 peptides. In response to defined signals, BAX and BAK are activated and oligomerize, exposing its C-terminal membrane-anchoring domain, and inserting into mitochondrial membranes [59]. In the classical model, the anti-apoptotic members of the family such as BCL-2 or BCL-xL inhibit BAX and BAK activation through a direct interaction involving the so called BH domains (the BCL-2 homology domains, which define both the structural and the functional homology patterns within the BCL-2 family). In this model, BAX activation is favored by the pro-apoptotic BH3-only proteins. These proteins can act both as direct activators of BAX as BID, BIM or PUMA, or as de-repressors as BAD, NOXA that interact with anti-apoptotic members of the BCL-2 family [60]. In this work we could demonstrate that leptin treatment decreases BID protein. All these results consolidate the anti-apoptotic role of leptin in placental cells.

It was demonstrated that the human BAX gene promoter contains typical p53-binding sites and is transcriptionally upregulated by p53[12]. The p53 tumor suppressor protein is a key component of cellular mechanisms that are activated by cellular stresses [61]. Therefore, we investigated whether this key cell cycle-signaling protein was involved in the effect of leptin. We determined p53 expression in the presence or absence of leptin in a model of serum deprivation condition, both in Swan-71 and human placental explants. A significant decrease of p53 was observed in both models demonstrating that leptin regulates p53 level under stress. Under normal conditions, p53 is a short-lived protein that is highly regulated and maintained at low or undetectable levels. After stress, such us serum deprivation, p53 is activated mostly at the post-translational level by a complex series of modifications that include the phosphorylation and acetylation of specific residues in the amino-terminal and carboxy-terminal domains. In addition to post-translational modifications, protein–protein interactions and subcellular relocalization also have a role in the activation of p53 [62]. The activation of p53 leads to the transcription of several genes whose products trigger different biological outcomes (for example, cell cycle arrest, apoptosis, DNA repair, replicative senescence or differentiation). MDM2 is known to negatively regulate p53 by mediating its ubiquitination and subsequent degradation in the proteasome. Little is known about leptin effect on MDM2 levels, but we speculate that leptin might regulate MDM2 expression, triggering the degradation of p53. Our data demonstrated that leptin not only decreases p53 protein level but also p53 mRNA level, measured by *q*RT-PCR. These results will be further explore, studying the mechanisms by which leptin regulates p53 expression.

Not all of the pathways involved in the processes regulated by p53 are known but phosphorylation of p53 at Ser-46 was shown to be involved in the regulation of apoptosis after DNA damage. Moreover, there is evidence that Ser-46 of p53 is phosphorylated in response to DNA damage *in vivo*, and it plays a pivotal role for apoptotic signaling by p53 through regulating the transcriptional activation of an apoptosis-inducing gene, p53AIP1 [10]. We decided to study the effect of leptin on this post-translational modification. Our data demonstrated that leptin significantly diminishes Ser-46 of p53 under stress condition. All these results confirmed the involvement of p53 regulation in leptin anti-apoptotic effect.

Our findings provide evidence for an inhibitory effect of leptin on the cell apoptosis program, suggesting a trophic role of leptin in the physiology of trophoblast cells. In addition, we have provided some evidence for the possible anti-apoptotic mechanisms of the leptin produced by trophoblastic cells. However, further

additional studies are needed to fully explain the effect of leptin on the regulation of BCL-2, BAX and p53 expression. More precisely, we have demonstrated the autocrine anti-apoptotic effect of leptin in trophoblastic cells, providing new insights into the functions of leptin in placental apoptosis. Since apoptosis plays a central role in placental physiology, our work further support the importance of leptin in human placenta.
