# Correction: Leptin Is an Anti-Apoptotic Effector in Placental Cells Involving p53 Downregulation

**DOI:** 10.1371/journal.pone.0107632

**Published:** 2014-09-02

**Authors:** The PLOS ONE Staff

The fourth and seventh authors’ names are spelled incorrectly. The fourth author’s correct name is: Antonio Pérez-Pérez. The seventh author’s correct name is: Víctor Sánchez-Margalet. The correct citation is: Toro AR, Maymó JL, Ibarbalz FM, Pérez-Pérez A, Maskin B, et al. (2014) Leptin Is an Anti-Apoptotic Effector in Placental Cells Involving p53 Downregulation. PLoS ONE 9(6): e99187. doi:10.1371/journal.pone.0099187
